# Cross‐sectional study on the etiological diagnosis of the patients with chronic prostatitis‐like symptoms by application of the urine‐prostate‐semen test

**DOI:** 10.1002/hsr2.574

**Published:** 2022-04-01

**Authors:** Danni Wang, He Wang

**Affiliations:** ^1^ Laboratory of Bacterial Pathogenesis, Department of Microbiology and Immunology, Institutes of Medical Sciences Shanghai Jiao Tong University School of Medicine Shanghai Shanghai China; ^2^ Department of Microbiology Guizhou Medical University Guiyang Guizhou China

**Keywords:** diagnosis, drug‐resistance, multiorgan infection, multiple microbial infection, prostatitis, urine‐prostate‐semen test

## Abstract

**Background and Aims:**

The prostatic secretion was considered to be the most important and even only specimen in diagnosis and differential diagnosis of chronic prostatitis like symptoms, but little attention has been paid to other genital organ infections. A urine‐prostate‐semen test (U‐EPS‐S test) was used to investigate the microbial flora of internal genital organs in patients with chronic prostatitis‐like symptoms and their influence on the diagnosis and treatment of the patients.

**Materials and Methods:**

We randomly selected the patients with chronic prostatitis‐like symptoms for this study and used a U‐EPS‐S test to collect urine, prostatic secretion, and semen specimens. The specimens were inoculated respectively into a suitable culture medium for bacteriological/fungal detection, and the number and distribution of colonies in each isolation culture were observed before and after the therapy.

**Results:**

All of the specimens from the internal genital organs of these patients were shown as microbe‐positive isolation and the infection rate was 100%. Of these, prostatic secretion with microbe‐positive isolation was obtained in 66 cases (33%), semen with microbe‐positive isolation was obtained in 34 cases (17%), and both prostatic secretion and semen with microbe‐positive isolation were obtained in 100 cases (50%). In the isolates, Gram‐positive microbes were shown as the most common pathogens, accounting for 91.1%. In 200 patients, 95 patients were infected with one microbial species infection, of them 36 were prostatic secretion positive‐isolation (18%), 20 were semen sample positive‐isolation (10%), and 39 were positive‐isolation both prostatic secretion and semen samples (19.5%); 104 patients were infected with two microbial species, of them 30 were prostatic secretion positive‐isolation (15%), 14 were semen sample positive‐isolation (7%), and 60 were positive‐isolation both prostatic secretion and semen samples (30%); one patient was infected with three microbial species and them were isolated from the semen sample (0.5%). In the patients with chronic prostatitis‐like symptoms, the multiple microbial infection (MMI) was accounted for 53.5%, and the multiorgan infection (MOI) was accounted for 67%.

**Conclusions:**

The U‐EPS‐S test is not only helpful to accurately identify the pathogens and contaminants in the culture isolates, but also the diagnosis and differential diagnosis and also evaluation of the treatment efficacy of the infection in different genital organs. In the patients with chronic prostatitis symptoms, Gram‐positive microbes were the most common causative agents, and MMI and MOI caused by resistant strains of different microbial species have a high incidence.

## 1 INTRODUCTION

The term prostatitis is often used to describe a symptom complex rather than a specific disease entity. The NIH Classification System divided prostatitis into four distinct categories: acute bacterial prostatitis (Category I), chronic bacterial prostatitis (Category II), chronic pelvic pain syndrome (Category III), and asymptomatic inflammatory prostatitis (Category IV).^1^ Chronic bacterial prostatitis is one of the most common diseases in andrology; it can also cause some serious complications related to abnormal expression of cytokines and immune responses in the prostate.^2^, ^3^ For a long time, diagnosis for the patients was based primarily on clinical manifestations, which are known as chronic prostatitis‐like symptoms, digital rectal examination (DRE), cytological, and/or bacteriological tests of urine‐prostatic secretion.^4^ The Meares–Stamey four‐glass urine test and other urine tests are the most commonly used methods in the etiological diagnosis of patients with chronic prostatitis‐like symptoms; these tests find the Gram‐negative bacteria, especially *Enterobacteriaceae* spp., to be the most common causative agents.^4^, ^5^ However, the treatment of these patients is always unsatisfactory even when an antibiotic is selected according to the results of in‐vitro sensitivity tests of the isolation culture of prostatic secretion as most antimicrobials can not diffuse into the prostatic secretion.^6^ It also results in the empirical treatments with antimicrobial agents to become a common phenomenon in the therapy of the patients with chronic prostatitis‐like symptoms clinically.^7^ In recent years, however, it has been confirmed through animal experiments and clinical observations that the dye trypan blue and almost all kinds of antimicrobial drugs can diffuse into the prostatic tissue and prostatic secretion at sufficiently high concentrations.^8^, ^9^ Some studies have shown that the prostatic secretions of patients with chronic prostatitis‐like symptoms often contain different species of microorganisms or different microbial strains with distinct biological properties or drug sensitivities.^10^, ^11^ These suggest that the heterogeneity of biological properties of infecting bacteria, drug resistance of pathogens, and methods of specimen collection might be important factors that affect the treatment efficacy of chronic bacterial prostatitis. A urine‐prostate‐semen test (U‐EPS‐S test) was developed and applied in the etiological diagnosis of patients with chronic prostatitis‐like symptoms; it has been shown to be helpful in the diagnosis and differential diagnosis and of the infecting pathogens and the infecting organs in the patients. Here, we used the U‐EPS‐S test to further investigated the microbial flora and their distribution in the genital organs of patients with chronic prostatitis‐like symptoms and drug sensitivities, and explore the influence of them on the etiological diagnosis and treatment efficacy of the patients.

## 2 MATERIALS AND METHODS

### 2.1 Study participants

In total, 200 participants from the clinic services of hospitals in China were randomly selected. All of them were diagnosed with chronic prostatitis according to symptoms and/or the clinical cytological examination of prostatic secretions; however, a bacteriological/fungal test on prostatic secretions was not performed for any.

### 2.2 The U‐EPS‐S test

**Figure 1:**
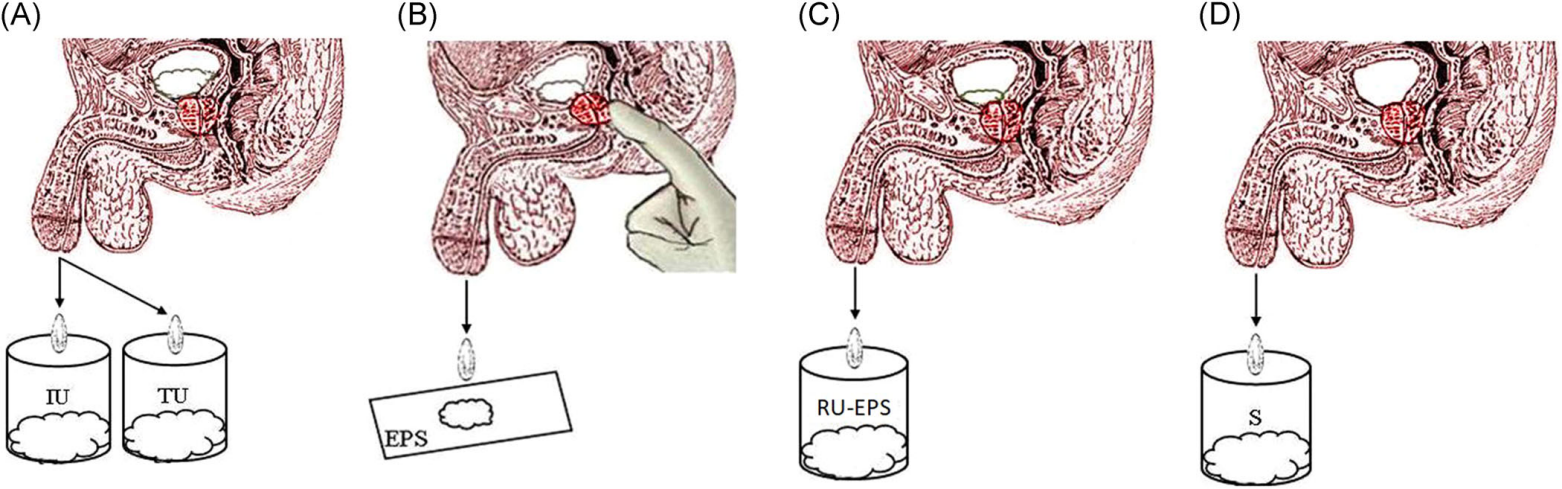
The urine‐prostate‐semen test is used for patients with prostatitis‐like symptoms. (A) The initial urinary stream (IU) and the third part of the urinary stream (TU) (1–10 ml each) were collected into different sterilized containers and used for microbiological testing. (B) By prostatic massage, the expressed prostatic secretion (EPS) overflowed from the urethral orifice onto a glass slide that was used for the routine test. (C) The EPS within the urethra was collected into a sterilized container by the patient urinating approximately 1–5 ml of residual urine (RU), and a mixture of EPS and RU (RU‐EPS) was used for the microbiological testing. (D) Semen collected by masturbation, sexual intercourse, or another method was used for microbiological and routine tests.

Specimens including segmented urinary stream (IU, TU), expressed prostatic secretion (EPS) and semen (S) were collected respectively by the U‐EPS‐S test from patients who had stopped the antimicrobial treatment for more than 3 days, the methods were shown in Figure 1.

### 2.3 Isolation of the pathogens

The specimens (0.1 ml) were inoculated onto blood agar, Sabouraud agar, and mycoplasma agar plates, and incubated under common air or 5% C_2_O conditions at 37°C.^12^, ^13^ The pathogens and contaminating microbes were distinguished according to the relative number of each kind of growth on each plate and their distribution in each isolation culture. The term “absolute number” refers to the total number of colonies formed by different microbial species on a medium; “relative number” refers to the total number of colonies formed by one microbial species on a medium and their comparison with the number of the same colonies on another medium; and “distribution” refers to the presence of certain microbes and their number in different culture isolates.

### 2.4 Identification of the isolates

The organism isolates from the specimens were identified respectively by routine bacteriological/fungal methods and/or gene assays.^13^, ^14^

### 2.5 Post‐therapy pathogen redetection

Antimicrobials were selected according to the results of in‐vitro sensitivity tests and other relevant necessary factors related to the patient and others, and the dosage and administration (oral or IV) of antimicrobial agents were according to the drug's instructions. In general, the course of treatment was 7–15 days (oral or intravenous administration) for patients with bacterial infection and 20–30 days (oral administration) for those with fungal infections.^9^ After the patients finished a course of treatment and had stopped using the antimicrobials for at least 3 days, the IU, TU, RU‐EPS, and S were collected again and used for pathogen detection according to the methods described above.

### 2.6 Statistical analysis

All statistical analyses were performed by SPSS (18.0) software (SPSS Inc.). A nonparametric Wilcoxon's‐sign rank test was used for data analysis. *p* < 0.05 was considered statistically significant. Statistical analysis was performed according to the following formulas.

Wilcoxon's‐sign rank test

$$Z=\frac{T-\mu_{T}}{\sigma_{T}}\mu_{T}=n(n+1)/4\sigma_{T}=\sqrt{n(n+1)(2n+1)/24}.$$

When the size of sample (*n*) is small:

$$Z=\frac{\left|T-\mu_{T}\right|-0.5}{\sigma_{T}}=\frac{\left|T-\frac{n\left(n+1\right)}{4}\right|-0.5}{\sqrt{n(n+1)(2n+1)/24}}.$$

When the rank has many ties:

$$Z_{t}=\frac{\left|T-\frac{n\left(n+1\right)}{4}\right|-0.5}{\sqrt{\frac{n\left(n+1\right)\left(2n+1\right)}{24}}-\frac{\sum \left(t_{j}^{3}-t_{j}\right)}{48}}.$$

## 3 RESULTS

### 3.1 General information of the study participants

The age range of the study participants was 20–85 years (mean age = 46.9 years), including 20–30 years old (56/200, 28%), 31–40 years old (58/200, 29%), 41–50 years old (37/200, 18.5%), 51–60 years old (33/200, 16.5%), 61–70 years old (10/200, 5%), and 71–85 years old (6/200, 3%). All of them had been diagnosed with chronic prostatitis based only on their symptoms and/or the cytological examination of prostatic secretions, but the bacteriological/fungal test for their prostatic secretions and semen or even urine never be performed for any. All of the study participants received antimicrobial agent (antibiotics and synthetic antimicrobial drugs) and/or the traditional Chinese medicine empirical treatments respectively. The course of treatment continued on and off for more than 6 months and in some of them even up to 20 years; however, their symptoms were not relieved or they had recurrent attacks.

### 3.2 Isolates and their diagnostic value

**Figure 2:**
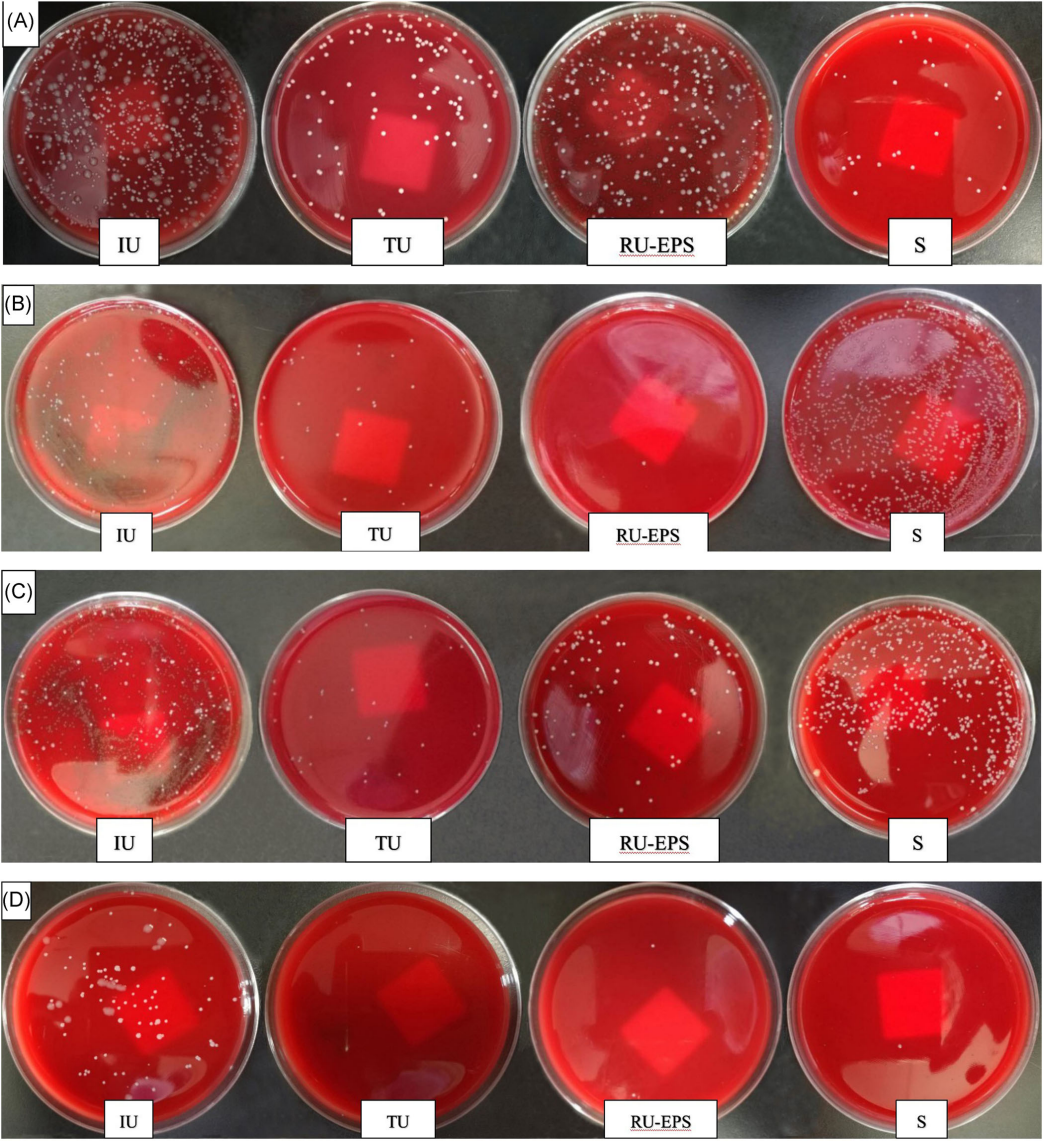
The diagnostic value of culture isolates in samples from patients with prostatitis‐like symptoms. The part label (A) shows that the absolute number of colonies was relatively large in the IU culture; the colonies consisted of at least three kinds of different bacterial species. In the TU culture, the absolute number of colonies was significantly lower; in the residual urine‐expressed prostatic secretion (RU‐EPS) culture, the absolute number of colonies was significantly increased. In the RU‐EPS culture, at least three kinds of colonies were significantly reduced. In the S culture, only one kind of colony was present. This patient can be diagnosed with multiple microbial infections (MMIs) of the prostate according to the relative number of colonies of each bacterial species and their distribution characteristics in each culture. The part label (B) shows that the absolute number of colonies was relatively large in the IU culture, in which at least two kinds of colonies; that in the TU culture was significantly reduced, with only one kind of colony, and that in the RU‐EPS culture was significantly reduced or absent. The absolute number of colonies in the S culture was significantly increased, as were the absolute number and a relative number of colonies, of which the relative number of colonies contained only one kind of colony. This patient can be diagnosed as the infections of deferens tract and/or other internal genital organs but not prostate infection. The part label (C) shows that the absolute number of colonies was relatively large in the IU culture with at least two kinds of colonies, that in the TU culture was significantly reduced, with only one kind of colony, and that in the RU‐EPS culture was significantly increased compared to that in the TU culture, with at least two kinds of colonies. The absolute number of colonies in the S culture was also significantly increased, with at least two kinds of colonies diagnosed as both prostate and deferens tract and/or other internal genital organ infection and MMI and multiorgan infection of the internal genital organs. The part label (D) shows that the absolute number of colonies was relatively large in the IU culture, with at least three kinds of colonies; the absolute number and number of colonies in the TU culture, the RU‐EPS culture, and the S culture were significantly reduced or absent. This patient can be diagnosed with no infection of the prostate or other internal genital organs; the results can also indicate that the patient has been cured if it is the result of posttherapy pathogen detection.

**Figure 3:**
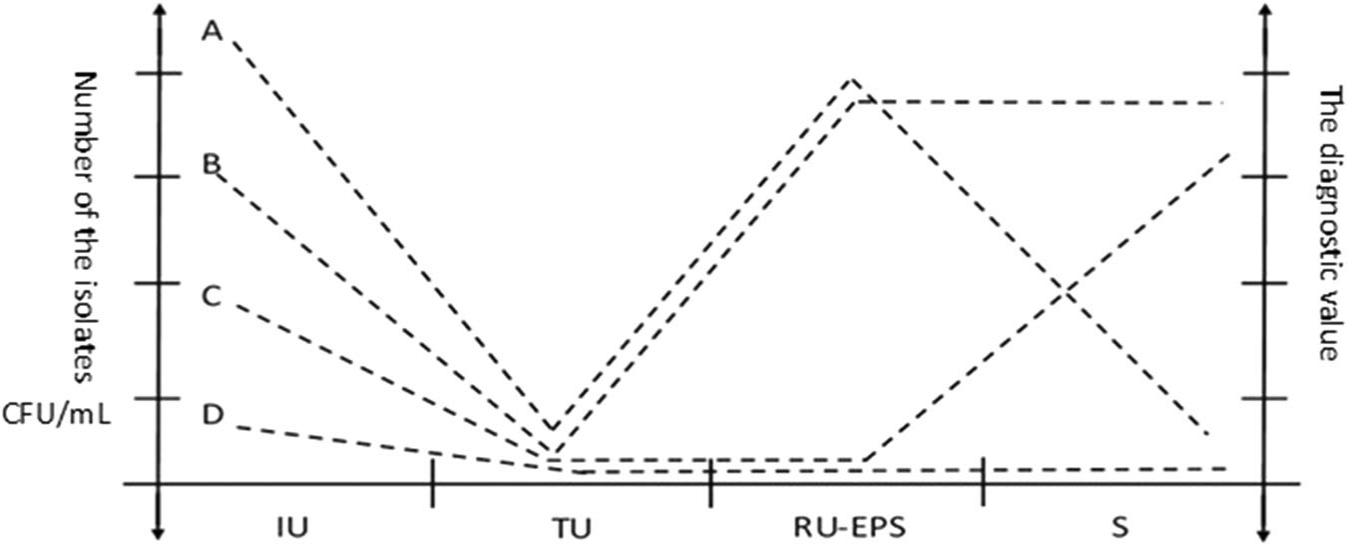
The curve of culture isolates from various specimens and the diagnostic value in etiology. (A) Simple prostate infection; (B) infection of the prostate and the other genital organs; (C) the prostate without infection but the other genital organs infection; (D) the prostate and the other genital organs without infection; (A–C) also had an infection of the lower urinary tract but not the upper urinary tract.

Number and distribution characteristics of the microbes on each culture media could be clearly observed and distinguished by naked eyes, and their species could be further identified by Gram stain and microscopy (Figure 2). If the number of colonies (CFU/ml) was determined and plotted, a curve of the type shown in Figure 3 was usually obtained, it could be sued to indicate the absolute number and relative number of the isolates and their distribution in various specimen. For example, according to the Figures 2 and the Figure 3, it shown that the absolute number of colonies (CFU/ml) in the IU culture is large, whereas the absolute number of colonies (CFU/ml) in the TU culture is significantly decreased because the washing and dilution of the urine stream. The relative number of colonies in TU is significantly decreased and obviously less than that in IU; it indicates the patient without upper urinary tract infection (such as the TU and IU cultures of Figure 2A,B,C,D, and the TU and IU curve of Figure 3A,B,C,D). If the infection was only occurred in patient is prostate, then the absolute number and relative number of colonies in RU EPS would be increased and obvious high than that in TU and semen (such as the cultures of Figure 2A and the curve of Figure 3A). If the infection was occurred in the other internal genital organs but not the prostate, the absolute number and relative number of colonies in semen would be significantly increased and obvious less than that in TU and RU EPS (such as the cultures of Figure 2B and the curve of Figure 3B). If the patient was the infections of prostate and other internal genital organs, the absolute number and relative number of colonies in RU EPS and semen would be significantly increased and obvious higher than that in TU (such as the cultures of Figure 2C and the curve of Figure 3C). If it was only the IU with microbe positive isolation but TU, RU EPS, and semen to be microbe negative isolation, it indicated the prostate and other internal genital organs without infection, it could also be used to indicated that the other genital organ infections of patient have had been cured (such as the cultures of Figure 2D and the curve of Figure 3D).

Based on the absolute number and the relative number of various growths and their distribution characteristics, the specimens, RU‐EPS or semen or both, from the internal genital organs of patients were shown as microbe‐positive isolation, the infection rate was 100% (200/200). Of which, 66 cases were the RU‐EPS with microbe‐positive isolation but the semen with microbe‐negative isolation, accounting for 33% (66/200); 34 cases were the semen with microbe‐positive isolation but RU‐EPS with microbe‐negative isolation, accounting for 17% (34/200); and 100 cases were microbe‐positive isolation of both the RU‐EPS and semen, accounting for 50% (100/200). Of the patients, 134 cases (34 semen with microbe‐positive isolation, 100 RU‐EPS, and semen with microbe‐positive isolation) were the multiorgan infection (MOI) of the genital tract and accounted for 67% (134/200).

Identification through routine bacteriological methods, a total of 468 strains of microorganisms were from the RU‐EPS and semen specimens of 200 patients, where most of the isolates isolated were Gram‐positive bacteria (91.1%, 377/468) (Table 1). Of the patients, 95 cases were infected with one microbial species, of them 36 were prostatic secretion positive‐isolation (18%, 36/200), 20 were semen sample positive‐isolation (10%, 20/200), and 39 were positive‐isolation of prostatic secretion and semen samples (19.5%, 39/200); 104 cases were infected with two microbial species, of them 30 were prostatic secretion positive‐isolation (15%, 30/200), 14 were semen sample positive‐isolation (7%, 14/200), and 60 were positive‐isolation of prostatic secretion and semen samples (30%, 60/200); one case was infected with three microbial species which was isolated from the semen sample (0.5%, 1/200). In the patients with chronic prostatitis‐like symptoms, the multiple microbial infection (MMI) was accounted for 53.5% (107/200). The drug sensitivity test in‐vitro showed that the bacterial isolates had different drug sensitivities, especially the resistance to the β‐lactam antibiotics and quinolones (Table 2).

**Table 1:** Isolates from the internal genital organs of patients with chronic prostatitis‐like symptoms

Category of microorganism (isolation rate)		Genus or family	The species identified by routine bacteriological/fungal methods (isolation rate, %)
Bacteria (88.5%, 414/468)	Gram‐positive (91.1%, 377/414)	*Staphylococcus* (64.2%, 242/377)	*S. Epidermidis* (46.7), *S. aureus* (3.7), *S. hominis* (4.0*), S. cohnii* (3.4*), S. capitis* (1.3), *S. saprophyticus* (1.9), *S. simulans (*1.3*), S. haemolyticus* (0.8), *S. intermedius* (0.3), *S. xylosus* (0.5*), S. wayneri* (0.3),
		*Streptococcus* (12.5%, 47/377)	β‐haemolytic streptococci (0.3), *S. faecalis* (9.5), *S. durans* (1.6*), S. faecium* (0.8), *S. bovis* (0.3),
		*Corynebacterium* (22.0%, 83/377)	*C. Psudodiphtheriae* (12.5), *C. xerosis* (6.6), *C. bovis* (1.3*), C. pseudotuberculosis* (0.5), *C. genitalium* (0.5), *C. equi* (0.3*), C. kutscheri* (0.3)
		Others (1.3%, 5/377)	*Lactobacillus spp*. (1.3)
	Gram‐negatives (8.9%, 37/414)	*Enterobacteriaceae* (62.2%, 23/37)	*Escherichia coli* (21.7), *Proteus vulgaris* (10.8), *Acinetobacter* spp. (8.1), *Moraxella* spp. (5.4), *Klebsiella pneumoniae* (5.4), *Citrobacter* spp. (5.4), *Enterobacter aerogenes* (2.7), *Pseudomonas aeruginosa* (2.7)
		*Neisseria* (37.8%, 14/37)	*N. Gonorrhoeae* (5.4), *N. mucosa* (8.1), *N. cinerea* (8.1), *N. subflava* (5.4), *N. sicca* (5.4), *N. lactamica* (2.7), *N. polysaccharea* (2.7)
Fungi (2.6%, 12/468)	Yeast (75.0%, 9/12)	*Candida* (75.0)	*C. albicans* (33.4), *C. krusei* (25.0), *C. paapsilosis (*8.3*), C. stellatoidea* (8.3)
	Filamentous fungi (25.0%, 3/12)	‐	*Aspergillus flavus* (16.7), *Penicillium* spp. (8.3)
Mycoplasma (8.5%, 40/468)	‐	‐	*Ureaplasma urealyticum* (92.5), *Mycoplasma genitalium* (5.0), *Mycoplasma hominis* (2.5)
Chlamydia (0.4%, 2/468)	‐	‐	*Chlamydia* spp.

**Table 2:** Drug sensitivities of bacterial species from patients with chronic prostatitis‐like symptoms

Antibacterials	*Staphylococcus* spp.			*Enterococcus* spp.			*Corynebacterium* spp.			*Enterobacteriaceae* spp.			*Neisseria* spp.			Amount to		
	Tested strains (*n*=)	Sensitive strains (*n*=)	Drug‐resistant rate (%)	Tested strains (*n*=)	Sensitive strains (*n*=)	Drug‐resistant rate (%)	Tested strains (*n*=)	Sensitive strains (*n*=)	Drug‐resistant rate (%)	Tested strains (*n*=)	Sensitive strains (*n*=)	Drug‐resistant rate (%)	Tested strains (*n*=)	Sensitive strains (*n*=)	Drug‐resistant rate (%)	Tested strains (*n*=)	Sensitive strains (*n*=)	Drug‐resistant rate (%)
Piperacillin	17	0	100	9	1	88.9	4	0	100	1	0	100	0	0	0	31	1	96.8
Cefazolin	87	54	37.9	24	13	45.8	25	10	60	0	0	0	11	10	0.91'	147	87	40.8
Cefuroxime	71	36	49.3	23	13	43.5	23	12	47.8	5	0	100	10	6	40	132	67	49.2
Cefotaxime	10	0	100	10	3	70	7	1	85.7	2	0	100	10	6	40	39	10	74.4
Ceftriaxone	22	4	81.8	8	0	0	6	2	66.7	5	0	100	10	7	30	51	13	74.5
Cefoperazone/sulbactam	88	60	31.8	24	15	37.5	6	5	16.7	5	5	0	11	7	27.3	134	92	31.3
Amikacin	53	33	37.7	21	5	76.2	19	12	36.8	3	0	100	1	1	0	97	51	47.4
Ciprofloxacin	48	6	87.5	22	4	81.8	14	0	100	4	1	75	10	10	0	98	21	78.6
Levofloxacin	61	18	70.5	18	4	77.8	12	1	91.7	2	2	0	11	1	90.9	104	26	75
Fleroxacin	7	0	100	7	0	100	5	0	100	3	0	100	0	0	0	22	22	100
Lomefloxacin	5	0	100	0	0	0	4	0	100	1	0	100	0	0	0	10	10	100
Minocycline	73	60	17.8	23	18	21.7	20	15	25	5	2	60	11	8	27.3	129	103	20.2
Fosfomycin	84	41	51.2	29	11	62.1	20	3	85	5	2	60	11	5	54.5	149	62	58.4
Imipenem	55	51	7.3	23	21	8.7	17	15	11.8	4	4	0	10	10	0	109	101	7.3

### 3.3 Laboratory findings after antimicrobial therapy

After each treatment course, the absolute number (CFU) and species of the pathogens in the IU, TU, RU‐EPS, and semen specimens were significantly reduced or completely disappeared compared to the number before therapy, changes in the bacterial species did not occur unless it was severe histopathological damage in the genital organs or the patient was sexually unclean during therapy (Figure 2D and Table 3). However, the absolute number (CFU) of pathogen in some patients, especially the patients with deferens tract infection after the first course of therapy, would also be showed a significant increase than that before the treatment. In the cases, the pathogens isolated from their specimens were often the drug resistant strains or the patients who did not ejaculate for a long time. Through the drug sensitivity test in‐vitro, it was shown that the surviving pathogens or new isolates after each therapy were usually strains that are resistant to the antibiotic just used. It showed a significant reduction in the number of the pathogens in the specimens from most patients after the first course of therapy than that of before the therapy, and the statistical analysis of them showed the *p* < 0.0001 (Table 4).

**Table 3:** The absolute number of bacteria for 200 patients before and after the first course of therapy

Specimens	Before the first course of therapy			After the first course of therapy		
	Maximum number of colonies on a medium (CFU/ml)	Average number of colonies of all the cultures (CFU/ml)	Organism‐negative isolation (*n*=)	Maximum number of colonies on a medium (CFU/ml)	Average number of colonies of all the cultures (CFU/ml)	Organism‐negative isolation (*n*=)
IU	>100,000	5433.9	2	20,000	500.1	19
TU	80,000	2718.6	5	28,000	218.9	24
RU‐EPS	60,000	5484.8	66	8500	1862.5	78
Semen	>100,000	12,232.3	34	12,000	5193.8	47

Abbreviations: EPS, expressed prostatic secretion; RU, residual urine.

**Table 4:** Statistical analysis of the absolute number of culture isolates from the patients before and after the first course of therapy

Specimens	Before^a^	After^a^	*p*
IU (CFU/ml)	3000 (1500, 4600)	90 (20, 300)	<0.001
TU (CFU/ml)	800 (182.5, 2600)	40 (10, 120)	<0.001
RU‐EPS (CFU/ml)	1200 (0, 6000)	570 (0, 3350)	<0.001
Semen (CFU/ml)	4000 (280, 16,000)	4000 (10, 11,000)	<0.001

Abbreviations: EPS, expressed prostatic secretion; RU, residual urine.

^a^ Data are presented as M (P_25_, P_75_).

## 4 DISCUSSION

The patients with chronic prostatitis‐like symptoms often have similar clinical manifestations, so the diagnosis and differential diagnosis of patients with chronic bacterial prostatitis or chronic prostatitis caused by other pathogen infection can depend only on the isolation of causative agents from prostatic secretions. However, it is well known that the male urethra contains normal flora, and they inevitably seem to contaminate the prostatic secretion collected by DRE. The Meares–Stamey four‐glass urine test has been considered to be an important method to differentiate prostatitis‐pathogens from urethral contamination. The Meares–Stamey four‐glass urine test, same as other urine tests, only specimens of segmented urine (VB1, VB2, and VB3) and EPS are collected but not semen, the volume of VB1, VB2, and VB3 are required to be more than 10 ml, respectively.^4^ This testing is informative only if urine from the bladder shows no bacterial growth, and any acute infection must first be treated. Thus, all of these tests often require the glans and penis of the patient to be cleaned with water and disinfected with a disinfecting agent. β‐lactam and nitrofurantoin have also been the most commonly used antimicrobial agents with prostatic specimen collection because it is considered that they do not penetrate the prostate.^4^ Same as the routine bacteriological methods, the diagnostic significance of isolates from Meares–Stamey four‐glass urine test and the other urine tests are mainly based on their absolute number and distribution; if isolates were found only in the prostatic fluid or the total amount of the isolates in the prostatic secretion was 10 times the levels found in the urethra, the patient could be diagnosed with chronic bacterial prostatitis.^4^, ^10^ In our work, we found that the absolute number of different specimens collected from patients with chronic prostatitis‐like symptoms would be influenced by many factors, such as the patient's water intake, urination frequency, sexual excitability and ejaculation, urinary tract hygiene, use of antibiotics, and glans penis disinfection, which often result in a decrease or increase in the absolute number of isolates in IU and TU specimens. The U‐EPS‐S test does not require the patients to take any antibiotic or disinfect the glans penis before the specimens are collected, it can effectively avoid the influence of the other factors on the results of microbe isolation culture according to the relative number and distribution of the isolates. In the U‐EPS‐S test, the number and distribution of growths are the most import indexes to evaluate the diagnostic value of microbe isolation cultures, especially the relative number of pathogens and their distribution in different cultures are more important diagnostic value. As it is well known that the infected organs of the male genital tract, same as other infected organs or tissues, contained more pathogens, these pathogens could be expelled or released from the diseased organ or tissue by extrusion or other factors. In the research, we used the U‐EPS‐S test to collect the specimens of urinary stream, prostatic secretion, and semen from the patient in sequence. Of them that the segmented urine (IU, TU) represented microbiologic flora of the lower urinary tract and the upper urinary tract respectively; the RU‐EPS specimen collected after the IU and TU represented microbiologic flora of the prostate; the semen (S) specimen was collected finally and it represented microbiologic flora of the other internal genital organs. If the patient did not be the upper urinary tract infection, number of the microbes in the TU specimen would be showed as a significant reduction than that of IU specimen or even negative isolation of microbes with the flushing and dilution of the urine stream. It is helpful to identify the isolates in the RU‐EPS specimen that came mainly from the prostate but not urine, the microbes in semen specimens came mainly from deferens tract and/or other internal genital organs but not the prostate. Although there were concerns that the volume of the specimens, the time of prostatic secretion and semen collection and the interval between the collection and so on would lead to a reduction in the number of microorganisms in the specimen, we found, in the S‐EPS‐S test, that the absolute number of the pathogens is variable but the relative number and distribution of them are always characterized. Generally, each course of treatment can decrease significantly the number and species of the drug‐sensitive strains and even completely eliminate them from the infected organs of patients, but there are some exceptions or special cases. In our previous research ^9^, ^12^ and this study, it was found that the absolute number of pathogen in some patients, especially in the patients with deferens tract infection undergone first course of the therapy, can also be increased significantly than that before the treatment, they were often the drug‐resistant strains. However, this would not affect the trend of the absolute number and species of pathogens to be gradually decreased, and the characteristics of relative number and distribution of them in various specimens. Through the further standardized treatment, the pathogens in the genital organs of patients will be cleared completely and the patients will also be cured.^9^, ^12^ Therefore, the relative number and distribution of the microbes in the specimens are not only helpful for accurate diagnosis and differential diagnosis of the pathogens and the infected organ but also the evaluation of therapeutic effect on etiology.

According to some reports, the Gram‐negative bacteria, especially *Escherichia coli*, cause approximately 75%–80% of episodes in patients with chronic bacterial prostatitis.^4^, ^5^, ^6^, ^7^ Nonetheless, by these reports, even when the patients are treated with antibiotics based on the results of drug sensitivity tests in‐vitro of isolates, the response rates of antimicrobial therapy are still unsatisfactory. For this reason, the drug permeability of the prostate has been considered to be the most important factor affecting the therapeutic effect of prostatitis. In our previous studies, it was confirmed that almost various kinds of antibiotics can diffuse into the normal prostatic tissue and the prostatic tissue with chronic bacterial inflammation and/or BPH of experimental rats as well as the prostatic tissue and secretions of patients with chronic bacterial prostatitis and/or BPH; these different antimicrobial agents can reach sufficiently high concentrations in prostatic tissues and secretions of animals and patients and kill the drug‐sensitive strains of microbes, thus improving the inflammatory response of animal prostate tissues and curing the prostatitis of patients.^15^, ^16^ In this study, by using the U‐EPS‐S test, it was shown that most of the strains isolated from the genital organs of patients with chronic prostatitis‐like symptoms were Gram‐positive bacteria, especially coagulase‐negative *Staphylococcus* species. Some causative agents that were once thought to be male genital‐non‐pathogenic organisms, such as *Corynebacterium* and *Neisseria* species, were also successfully isolated and identified from the prostate and/or other internal genital organs by the U‐EPS‐S test. The diagnostic significance of the various isolates and their relationship with prostatitis or other genital infections can also be further determined by etiological and routine cytological examination after the therapy. According to the previous studies^9^ and this study, standardizing the use of antimicrobial agents to treat patients under the guidance of correct etiological examination results, the number and species of drug‐sensitive pathogens in the prostate and other genital organs should be significantly reduced and even cleaned. The patient's symptoms will also be significantly improved as the number of bacteria decreases, this is just the main reason why the patient is willing to see the doctor again and continue the next treatment. Accordingly, if a good response is not observed of the antimicrobial therapy according to the etiological test results of prostatic secretion, it usually indicates that the etiology examination is incorrect or the therapeutic method is not standard.

In this study, these patients with chronic prostatitis‐like symptoms have been diagnosed respectively as “chronic prostatitis,” “chronic bacterial prostatitis,” or “chronic pelvic pain syndrome” based on their clinical manifestations and treated by different antimicrobial agents. Unfortunately, however, the antimicrobial therapy for them did not base on the drug sensitivity test in‐vitro of the pathogens but on the experience of doctors. According to our previous research^12^, ^13^ and this study, it showed that the empiric antimicrobial therapy could not reduce the number and species of pathogens in the genital organs of these patients, the surviving pathogens were not only were abundant but also were often the strains with drug‐resistant or even multi‐resistant because of the selection of antimicrobial agents. In our studies, it was found on the basis of laboratory examinations that the number of drug‐sensitive bacteria was significantly reduced or even disappeared in the prostate gland of almost all patients after the fifth day of antimicrobial agent treatment intravenously or after the 9th day orally.^16^ Accordingly, based on these findings, we recommend that the usual course of antimicrobial therapy for patients with chronic bacterial infection of genital organs be 9–12 days orally or 6–8 days intravenously; for fungal infection, the course should be 20–30 days orally because of the biological properties of these organisms.^9^, ^12^ Effective treatment for chronic bacterial prostatitis should completely kill and remove the causative agents from the prostate rather than just improving the symptoms of patients. In other words, because MOI is very common in patients with chronic prostatitis‐like symptoms, the term “effective treatment” for patients with chronic prostatitis‐like symptoms should at least include both complete disappearances of the causative agents from the prostate and other internal genital organs and significant improvement of symptoms. Based on our past research and the findings of this study, bacteria with varying amounts may be left in the prostate after the end of each course of therapy, and these residues are often drug‐resistant strains. The microbes remaining in the prostate, even in small numbers, often cause acute or chronic recurrence of prostatitis after 6 months.^16^ Animal tests showed that abscesses or other pathological reactions can form within prostatic tissues because of bacterial infection; the pathogens held in pathological tissues can also be released because of extrusion of the prostate gland or the attenuated inflammatory reaction.^15^ This seems to explain why the number of pathogens in the RU‐EPS or semen specimens of some patients just treated with antimicrobial agents can be higher than that before treatment or why a new pathogen can be found. Therefore, pathogen redetection for the infected organ of the genital tract of patients after each course of treatment is very important, this is also an important indicator to evaluate the therapeutic effect. Further treatment or another course of treatment for these patients with microbe‐positive culture must be based on the results of new laboratory tests and drug sensitivity testing in‐vitro, and antimicrobial agents should never be used empirically. Based on these findings, it can be considered that nonstandard sample collection and incorrect etiologic diagnosis as well as the biodiversity and drug resistance of pathogens are the most important factors that render the treatment of chronic bacterial prostatitis‐like symptoms unsatisfactory.

There are three major limitations in this study that should be addressed in future research. First, it should highly focus on the relative number of each microbial species and their distribution in the isolation cultures, this is very important to distinguish between the pathogens and the contaminants in the specimens. Second, MMI and MOI caused by the drug‐resistant strains of different microbial species have a high incidence in the patients with chronic prostatitis‐like symptoms, so the IU, TU, EPS, and semen specimens should be collected systematically and sequentially from the patient, and paid high attention to each growth in the isolation cultures. Third, during the course of therapy, the number and species of the pathogens in the specimens collected from most patients will be decreased significantly with the therapy. The surviving pathogens after each course of treatment, even the same microbial species, are often the strains with single drug resistant or even multidrug resistant, so the post therapy pathogen redetection is very important or indispensable.

## 5 CONCLUSION

The results in this study confirmed: (i) The U‐EPS‐S test is not only helpful to accurately identify the pathogens and contaminants in the culture isolates but also the diagnosis and differential diagnosis and also evaluation of the treatment efficacy of the infection in different genital organs; (ii) In the patients with chronic prostatitis‐like symptoms, Gram‐positive microbes were the most common causative agents; (iii) MMI and MOI caused by resistant strains of different microbial species have a high incidence in the patients with chronic prostatitis‐like symptoms.

## AUTHOR CONTRIBUTIONS

**Danni Wang**: conceptualization; investigation; methodology; writing – original draft; writing – review & editing. **He Wang**: conceptualization; formal analysis; funding acquisition; investigation; methodology; project administration; resources; supervision; writing – original draft; writing – review & editing. All authors have reviewed the manuscript and approved submission. He Wang had full access to all of the data in this study and takes complete responsibility for the integrity of the data and the accuracy of the data analysis; that no important aspects of the study have been omitted; and that any discrepancies from the study as planned (and, if relevant, registered) have been explained.

## CONFLICTS OF INTEREST

The authors declare no conflicts of interest.

## ETHICS STATEMENT

This protocol was evaluated and approved by the ethics committee of Guizhou Medical University (Ethics Committee approval number: 2021–166). This study was in compliance with the Declaration of Human Rights, Chinese Ministry of Health's “Methods for Ethical Review of Biomedical Research Involving Human Beings (Trial),” and the relevant provisions of the Helsinki Declaration on biological human testing.

## Data Availability

We incorporated only peer‐reviewed, published articles. The datasets (as derived from the published papers) used and analyzed during the current study are available on reasonable request from the corresponding author.
